# Computed tomography-based virtual reality-guided preoperative simulation for endoscopic full-thickness resection of a gastric submucosal tumor

**DOI:** 10.1055/a-2445-8353

**Published:** 2024-11-18

**Authors:** Takeshi Uozumi, Seiichiro Abe, Maki Sugimoto, Mitsunori Kusuhara, Yasuhiko Mizuguchi, Satoru Nonaka, Yutaka Saito

**Affiliations:** 1 68380 Endoscopy Division, National Cancer Center Hospital, Tokyo, Japan; 2 Innovation Lab, Teikyo University Okinaga Research Institute, Tokyo, Japan

Virtual reality is gaining attention as a novel modality for precisely identifying the location of lesions and the routes of important vessels before and during surgery 1 2 . Endoscopic full-thickness resection (EFTR) is a minimally invasive treatment for gastric submucosal tumors (SMTs); however, owing to the nature of EFTR, with an intra-to-extraluminal blind approach, it carries the potential risk of damaging extraluminal vessels or organs. Virtual reality might help endoscopists accurately identify important vessels and anatomical structures during EFTR, facilitating the safe removal of SMTs. This is the first case report of computed tomography (CT)- and virtual reality-guided preoperative simulation of gastric EFTR.

A 41-year-old woman was diagnosed with a gastric SMT and was referred to our hospital. She had no underlying disease or history of abdominal surgery. The gastric SMT was located at the anterior wall of the lower gastric body, and was less than 30 mm without ulceration; therefore, we planned EFTR for this lesion.

Before EFTR, polygons (standard triangulated language format) of the stomach, artery, and SMT were created using data from DICOM (National Electrical Manufacturers Association, Rosslyn, Virginia, USA) from three-phase contrast-enhanced CT images. The polygons were uploaded to the Holoeyes MD system (Holoeyes Inc., Tokyo, Japan) and converted into virtual three-dimensional (3D) models 3 . We checked the arterial route and anatomical structure using a virtual reality head-mounted display ( Fig. 1 ) and found no major extraluminal artery around the SMT ( Fig. 2 ).

**Fig. 1:**
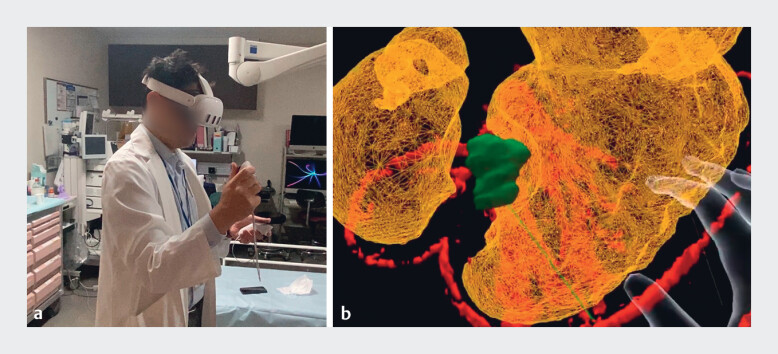
Preoperative virtual three-dimensional model. **a** Virtual reality can be experienced using a head-mounted display (Metaquest 3; Meta Platforms, Inc., Menlo Park, California, USA). **b** Model output: yellow, stomach; green, submucosal tumor; Red, artery.

**Fig. 2:**
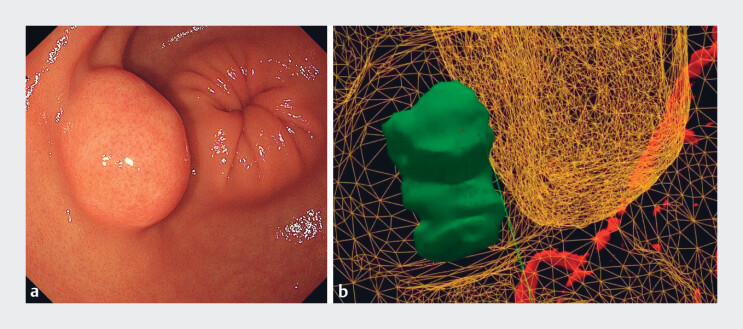
Endoscopic image and virtual three-dimensional model. **a** A 30-mm submucosal tumor was located in the anterior wall of the lower gastric body. **b** The submucosal tumor and the extraluminal artery could be observed inside the stomach using virtual reality imaging.

EFTR was safely completed without any intraoperative adverse events ( Video 1 ). No major extraluminal arteries were found during EFTR, as confirmed preoperatively. We closed the full-thickness defect using the endoloop–endoclip and reopenable-clip over-the-line methods. The pathological diagnosis was a low-risk gastric intestinal stromal tumor in the Modified Fletcher classification, with free lateral and deep margins.

Preoperative three-dimensional model with virtual reality and endoscopic full-thickness resection.Video 1

This case demonstrates preoperative simulations using CT-based virtual reality. Traditionally, the 3D reconstruction of CT images on a flat display has not provided adequate spatial understanding. However, by immersing ourselves in virtual reality, we could comprehend the spatial relationships between the gastric wall, tumor, and surrounding arteries. This immersive virtual reality experience significantly enhanced spatial awareness during the endoscopic procedures.

Endoscopy_UCTN_Code_TTT_1AO_2AG_3AF

## Notes

Endoscopy E-Videos https://eref.thieme.de/e-videos
*E-Videos* is an open access online section of the journal *Endoscopy* , reporting on interesting cases and new techniques in gastroenterological endoscopy. All papers include a high-quality video and are published with a Creative Commons CC-BY license. Endoscopy E-Videos qualify for HINARI discounts and waivers and eligibility is automatically checked during the submission process. We grant 100% waivers to articles whose corresponding authors are based in Group A countries and 50% waivers to those who are based in Group B countries as classified by Research4Life (see: https://www.research4life.org/access/eligibility/ ). This section has its own submission website at https://mc.manuscriptcentral.com/e-videos .
